# Avaliação da Acurácia do Escore ADHERE em uma Coorte Brasileira Internada por Insuficiência Cardíaca

**DOI:** 10.36660/abc.20250031

**Published:** 2025-12-18

**Authors:** Bruno Reznik Wajsbrot, Ana Luiza Ferreira Sales, Andre Luis Sales Feitosa, Carolina Pereira de Barros, Daniel Xavier de Brito Setta, Felipe Neves de Albuquerque, Marcelo Imbroinise Bittencourt, Pedro Pimenta de Mello Spineti, Simone Offrede Rego, Denilson Campos de Albuquerque, Roberto Esporcatte, Ricardo Mourilhe-Rocha

**Affiliations:** 1 Universidade do Estado do Rio de Janeiro Rio de Janeiro RJ Brasil Universidade do Estado do Rio de Janeiro, Rio de Janeiro, RJ – Brasil; 2 Hospital Pró-Cardíaco Rio de Janeiro RJ Brasil Hospital Pró-Cardíaco, Rio de Janeiro, RJ – Brasil; 3 Hospital Unimed-Rio Rio de Janeiro RJ Brasil Hospital Unimed-Rio, Rio de Janeiro, RJ – Brasil; 4 Complexo Hospitalar Américas Hospitais Vitória e Samaritano Barra Rio de Janeiro RJ Brasil Complexo Hospitalar Américas – Hospitais Vitória e Samaritano Barra, Rio de Janeiro, RJ – Brasil

**Keywords:** Insuficiência Cardíaca, Prognóstico, Mortalidade

## Abstract

**Fundamento:**

Pacientes hospitalizados por insuficiência cardíaca apresentam alta taxa de mortalidade, tornando a estratificação de risco para óbito de extrema importância. Os escores de risco podem ajudar a identificar pacientes com maior risco, mas é de suma importância validá-los na população que será utilizado, uma vez que as características sociodemográficas podem ser bastante heterogêneas entre diferentes populações, prejudicando sua acurácia.

**Objetivos:**

O objetivo deste estudo foi avaliar o desempenho do escore ADHERE *(Acute Decompensated Heart Failure Registry)* em um hospital universitário no Brasil.

**Métodos:**

Estudo tipo coorte retrospectivo envolvendo 304 pacientes com insuficiência cardíaca descompensada e idade ≥ 18 anos, realizado entre setembro de 2019 e julho de 2022. O desfecho primário foi a avaliação do escore ADHERE por meio da análise do índice discriminatório e classificação. O desfecho secundário foi a avaliação de outros fatores prognósticos para mortalidade hospitalar. O valor de p<0,05 foi considerado estatisticamente significativo.

**Resultados:**

O escore ADHERE apresentou índice discriminatório de 0,69. A capacidade de classificação do escore ADHERE foi subótima, pois o escore não estratificou o risco de mortalidade em cinco estratos, como fora proposto. Além disso, o escore subestimou o risco na população estudada. A ureia sérica na admissão foi o único fator prognóstico isolado para o desfecho (OR 1,043; IC 95% 1,024-1,062; p<0,001).

**Conclusão:**

O escore de risco ADHERE não pôde ser completamente validado em nossa coorte, uma vez que a classificação não foi alcançada. A ureia sérica na admissão foi o único fator de risco independente associado à mortalidade hospitalar. Nosso estudo enfatiza a importância da completa validação externa de um escore prognóstico, especialmente quando as características demográficas e clínicas das coortes não são comparáveis.

## Graphical abstract

**Figura Central:**
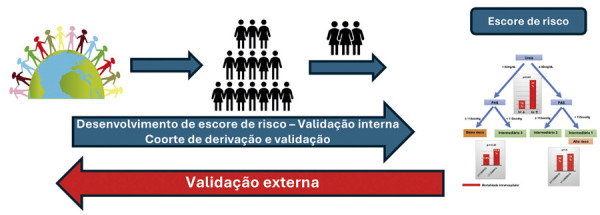
Avaliação da Acurácia do Escore ADHERE em uma Coorte Brasileira Internada por Insuficiência Cardíaca

**Figura Central:** Avaliação da Acurácia do Escore ADHERE em uma Coorte Brasileira Internada por Insuficiência Cardíaca

Fonte: o autor.

## Introdução

As doenças cardiovasculares são a principal causa de morte prematura no mundo.^1^ A insuficiência cardíaca (IC), um dos principais representantes desse grupo, pode ser considerada uma doença epidêmica não infecciosa,^2^ afetando cerca de 26 milhões de pessoas.^3^ Nos últimos dez anos no Brasil, as doenças cardiovasculares foram a segunda causa mais prevalente de morte, sendo a IC uma das principais causas específicas dentro deste grupo.^4^

A insuficiência cardíaca descompensada (ICD) é uma complicação grave da síndrome e é definida pela Sociedade Europeia de Cardiologia como “início rápido ou gradual de sintomas e/ou sinais de IC, suficientemente graves para que o paciente procure atendimento médico urgente”.^5^ A mortalidade hospitalar (MH) pode chegar a 20%, dependendo das características dos pacientes e da gravidade do quadro.^6^ A média da MH na Europa e nos EUA é de 4,9%^7^ e 4,0%,^6^ respectivamente, enquanto a maior coorte de ICD no Brasil, o estudo BREATHE (I Registro Brasileiro de Insuficiência Cardíaca), mostrou um valor de 12,6%.^8^ Para prever o risco de óbito hospitalar em pacientes hospitalizados por ICD, a Diretriz da Sociedade Americana de Cardiologia (*American Heart Association)* de 2022 para o Manejo da Insuficiência Cardíaca^9^ sugere o uso do escore ADHERE *(Acute Decompensated Heart Failure Registry).*^6^

Existe, no entanto, grande variabilidade nas características sociodemográficas entre diferentes populações e os fatores prognósticos podem variar entre os grupos. Por exemplo, a hipertensão isolada não associada a doença isquêmica do coração está fortemente associada à IC na África Subsaariana e na América Latina, mas não em países desenvolvidos, onde a etiologia isquêmica prevalece.^10^ No estudo BREATHE, a prevalência de miocardiopatia isquêmica no Brasil variou de 16,5% a 33,6%, dependendo da região estudada^8^. Os escores de risco de mortalidade devem ser capazes de discriminar a gravidade da doença, permitindo a correta alocação de recursos durante o tratamento. Para alcançar esse objetivo, a validação externa dos escores é fundamental^11^(Figura Central).

Embora as sociedades de cardiologia incentivem o uso do escore ADHERE em pacientes com ICD, uma validação completa usando todos os critérios estatísticos raramente é realizada.^12-14^ O objetivo deste estudo foi avaliar o desempenho do escore ADHERE em uma população brasileira de um hospital quaternário universitário. Os objetivos secundários foram a avaliação de outros fatores prognósticos, dados demográficos e clínicos, e sua relação com a MH.

## Métodos

Foi realizado estudo retrospectivo longitudinal unicêntrico. Os dados foram coletados de registros médicos eletrônicos entre setembro de 2019 e julho de 2022. Foram incluídos pacientes admitidos com diagnóstico de ICD, de acordo com a definição de 2021 da Sociedade Europeia de Cardiologia.^5^ Os critérios de exclusão foram idade inferior a 18 anos, diagnóstico prévio de malignidade em cuidados paliativos ou pacientes em terapia de substituição renal antes da internação.

Foram coletados dados clínicos e demográficos além de frequência cardíaca e pressão arterial de admissão. Os dados laboratoriais incluíram avaliação de sódio e potássio séricos na admissão, hemoglobina sérica, ureia e creatinina séricas de admissão, glicose aleatória sérica e hemoglobina glicada. A insuficiência renal foi considerada quando a taxa de filtração glomerular estimada (TFGe) estava abaixo de 60 ml/min/m^2^.^15,16^ A presença de diabetes mellitus foi considerada se autorrelatada ou se o paciente apresentasse glicose aleatória ≥ 200 mg/dL ou hemoglobina glicada ≥ 6,5%.^17^ Os parâmetros ecocardiográficos foram extraídos do primeiro exame realizado após a admissão ou de um exame anterior, caso não houvesse informação do ecocardiograma realizado no hospital. Os parâmetros avaliados foram fração de ejeção do ventrículo esquerdo, descrição qualitativa da função ventricular esquerda e direita, diâmetro diastólico e sistólico final do ventrículo esquerdo, volume do átrio esquerdo, presença e quantificação de regurgitação aórtica e mitral.

## Análise estatística

Foi realizado o teste de Kolmogorov-Smirnov para verificar a normalidade da distribuição das variáveis contínuas. Para as análises descritivas, as variáveis contínuas foram expressas como médias (desvio padrão) para dados de distribuição normal ou medianas (intervalo interquartil) para dados assimetricamente distribuídos, ou números absolutos (proporções) para variáveis categóricas. As diferenças entre dois grupos foram examinadas pelo teste t para amostras independentes ou teste de Mann-Whitney para variáveis normais ou de distribuição não paramétrica, respectivamente. O teste qui-quadrado ou teste exato de Fisher foi utilizado para avaliação de diferenças entre os grupos no caso de variáveis categóricas. Análise de regressão logística binária foi realizada para investigar as variáveis independentemente associadas ao óbito hospitalar. A validação do escore foi feita pela análise do índice discriminatório (ID) e calibração.^18^ O escore ADHERE logístico, utilizando a equação publicada previamente, foi utilizado para a análise da área sobre a curva (ASC) da curva ROC, que é, por definição, o ID. A calibração foi feita comparando os eventos esperados e observados na coorte brasileira. Todos os testes foram bicaudais e o valor de p<0,05 foi considerado estatisticamente significativo. As análises foram realizadas com o software SPSS versão 21.0 (Chicago, EUA).

O estudo foi revisado e aprovado pelo Comitê de Ética em Pesquisa em Saúde da Faculdade de Ciências Médicas da Universidade do Estado do Rio de Janeiro, número 3.706.949.

## Resultados

458 internações descritas como miocardiopatia (CID 25.5), insuficiência cardíaca não especificada (CID 50.9) e insuficiência cardíaca congestiva (CID 50.0) foram incluídas. Dessas, 154 foram excluídas por se tratar de internações repetidas do mesmo paciente, resultando em 304 internações analisadas.

As variáveis demográficas, clínicas, ecocardiográficas e laboratoriais podem ser vistas na Tabela 1. A média de idade foi de 63,04 anos (DP±14,3). 56,6% eram do sexo masculino, 65,8% tinham hipertensão arterial, 33,3% diabetes mellitus, 30,6% apresentavam diagnóstico prévio de fibrilação ou flutter atrial, e 30,9% apresentavam doença arterial coronariana, associada ou não à causa da IC; 75,5% foram classificados como insuficiência cardíaca crônica descompensada e 24,5% como insuficiência cardíaca de início agudo. A fração de ejeção foi medida em 274 pacientes (90,1%). Desses, 188 (68,6%) foram classificados como IC com fração de ejeção reduzida (ICFEr), 37 (13,5%) como IC com fração de ejeção levemente reduzida (ICFElr) e 49 (17,9%) como IC com fração de ejeção preservada (ICFEp). Do total avaliado, 156 (52,0%) apresentavam disfunção ventricular direita. A MH foi de 15,1% (46/304) para toda a coorte e 18,4%, 16,2% e 13,3% para os subgrupos de ICFEp, ICFElr e ICFEr, respectivamente (p=0,64).

**Tabela 1:** – Variáveis demográficas, clínicas, ecocardiográficas e laboratoriais da coorte e subgrupos

Variável	Total	Alta	Óbito	p-valor
	n=304	n=258 (84,9%)	n=46 (15,1%)	
Idade, anos	63,04 (±14,3)	62,22 (±14,1)	67,59 (±15,07)	0,02
Sexo masculino, n (%)	172 (56,6%)	152 (58,9%)	20 (43,5%)	0,052
IC isquêmica, n (%)	92 (30,9%)	80 (31,5%)	12 (27,3%)	0,724
Hipertensão, n (%)	198 (65,8%)	167 (65,2%)	31 (68,9%)	0,734
DM, n (%)	102 (33,7%)	81 (31,9%)	20 (43,5%)	0,126
Insuficiência renal, n (%)	65 (21,7%)	54 (21,2%)	11 (24,4%)	0,624
FA ou Flutter atrial, n (%)	92 (30,6%)	74 (28,9%)	18 (40%)	0,136
DPOC, n (%)	19 (6,3%)	18 (7%)	1 (2,2%)	0,327
Tabagismo, n (%)	58 (19,1%)	52 (20,2%)	6 (13%)	0,313
Etilismo, n (%)	38 (14,6%)	33 (14,9%)	5 (12,5%)	0,811
IC conhecida, n (%)	181 (75,7%)	153 (74,6%)	28 (82,4%)	0,394
Hemoglobina, mg/dL	13 (11,4-14,5)	13 (11,5-14,4)	12,1 (10,5-14,8)	0,429
Creatinina, mg/dL	1,4 (1,0-2,0)	1,38 (1,03-1,09)	1,7 (1,2-2,7)	0,003
TFG-e (ml/min/m^2^)	47 (23-75)	48 (24-76)	34 (16-71)	0,016
Sódio, mg/dL	136 (134-140)	137 (134-140)	135 (131-138)	0,008
Potássio, mg/dL	4,3 (3,8-4,8)	4,3 (3,8-4,8)	4,2 (3,8-4,8)	0,68
FC, bpm	81 (70-98)	81 (70-98)	82 (74-94)	0,807
PAS, mmHg	118,5 (103-135)	120 (107-140)	110 (100-130)	0,037
PAD, mmHg	71 (62-85)	74 (63-85)	70 (60-80)	0,017
Ureia, mg/dL	51 (34,9-83,5)	49,0 (34-76,9)	83 (39,8-145,9)	0,001
FEVE, %	33 (24-45)	33 (24-45)	35 (25-48,7)	0,585
DDVE, mm	59 (±11,8)	59,3 (±11,14)	57,6 (±15,27)	0,503
DSVE, mm	49 (39-58)	49 (40-58)	48,5 (32,2-58)	0,574
Volume AE, mL/m^2^	53 (32,2-64)	51 (42-62,5)	59 (53,5-73,7)	0,001
IM grave, n (%)	49 (19,2%)	41 (18,9%)	8 (21,1%)	0,755
Disfunção de VD, n (%)	156 (52,0%)	128 (50,4%)	28 (60,9%)	0,203

IC: insuficiência cardíaca; DM: diabetes mellitus; FA: flutter atrial; DPOC: doença pulmonar obstrutiva crônica; TFG-e: taxa de filtração glomerular estimada; FC: frequência cardíaca; PAS: pressão arterial sistólica; PAD: pressão arterial diastólica; FEVE: fração de ejeção do ventrículo esquerdo; DDVE: diâmetro diastólico final do ventrículo esquerdo; DSVE: diâmetro sistólico final do ventrículo esquerdo; AE: átrio esquerdo; IM: insuficiência mitral; VD: ventrículo direito.

### Desfecho primário

**Figura 1:**
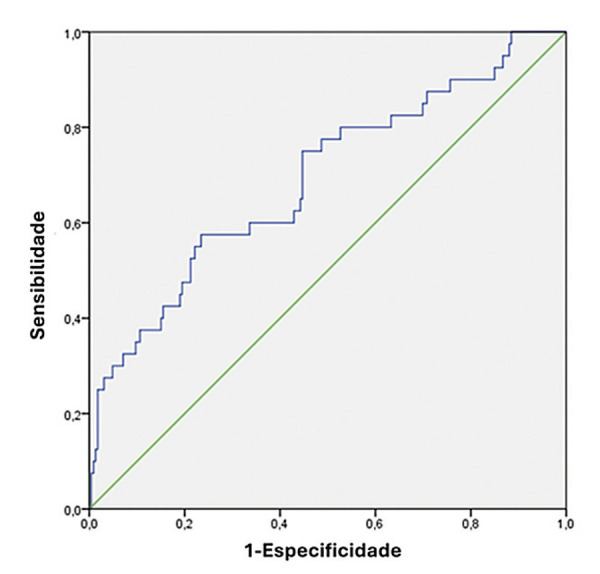
– Índice discriminatório do Escore ADHERE. Fonte: O autor.

**Figura 2:**
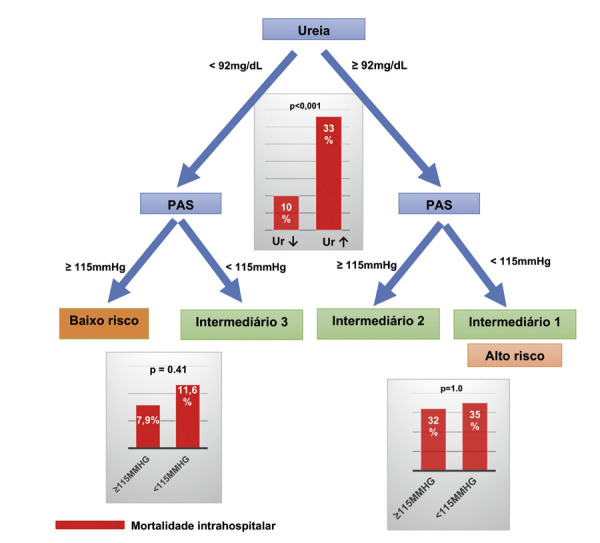
– Análise da classificação do Escore ADHERE. PAS: Pressão arterial sistólica. Fonte: O autor.

O escore ADHERE pôde ser calculado em 87,5% (266/304) dos pacientes. O ID do escore apresentou ASC de 0,69 (Figura 1). A classificação do escore ADHERE foi inadequada para prever a MH nos cinco grupos de risco propostos. Apenas a primeira divisão, representada pela ureia sérica na admissão, conseguiu criar dois grupos com diferentes mortalidades hospitalares (OR 4,45; IC 95% 2,29-8,65; p<0,001). O segundo preditor do escore ADHERE, representado pela pressão arterial sistólica (PAS), não classificou subgrupos conforme originalmente proposto. A análise de classificação pode ser encontrada na Figura 2.

### Desfechos secundários

As variáveis associadas à MH na análise univariada foram idade mais avançada (p=0,02), PAS reduzida (p=0,037), pressão arterial diastólica reduzida (p=0,017), maior concentração de ureia sérica na admissão (p=0,001), maior creatinina na admissão (p=0,003), menor sódio sérico de admissão (p=0,005) e maior volume do átrio esquerdo (p=0,008). O modelo exploratório com regressão logística binária, incluindo idade, PAS, ureia, TFGe e sódio sérico, mostrou que apenas a ureia foi independentemente relacionada à MH (OR 1,043; IC 95% 1,024-1,062; p<0,001). O ID da ureia foi de 0,65. A primeira divisão do escore ADHERE, representada por valores de ureia acima ou abaixo de 92 mg/dL, estratificou a população em subgrupos com MH de 33,3% e 10,1%, respectivamente.

## Discussão

Nosso estudo teve como objetivo avaliar o escore ADHERE em população brasileira de um centro universitário quaternário. O principal achado foi a incapacidade de validar completamente o escore proposto para prever a MH nesta população. A principal mensagem é que a validação de escores de risco antes de sua aplicação na prática clínica é imperativa. Embora sugeridos por diretrizes, os escores muitas vezes são imprecisos quando usados em populações diferentes das que os originaram.

Nossa coorte pertence a um centro de ensino e cuidados quaternários do sistema público de saúde brasileiro, especializado no tratamento de pacientes com IC avançada. Por outro lado, o ADHERE é derivado de um registro de mais de 65 mil pacientes admitidos em 263 hospitais dos EUA, entre 2001 e 2003. Nossa coorte representa provavelmente uma amostra de maior gravidade se comparada à população estadunidense que gerou o escore ADHERE e mesmo se comparada à população brasileira em geral, tratada em centros não especializados. A MH encontrada no estudo BREATHE^8^ foi menor do que em nossa coorte, mesmo em se tratando de uma avaliação de pacientes tratados em centros não especializados, mostrando provavelmente maior gravidade e características clínicas distintas da nossa população. A generalização dos nossos resultados para a população brasileira internada por ICD, portanto, não pode ser realizada.

A comparação entre nossa coorte e o registro ADHERE pode ser vista na Tabela 2. Foi encontrada média de idade quase dez anos mais jovem, com menores valores de pressão arterial na admissão, menor ureia e creatinina séricas do que a população do estudo ADHERE. Além disso, diabetes e hipertensão eram menos conhecidos. A distribuição dos fenótipos de IC também foi diferente entre as coortes.^19^ Tivemos mais de 70% de ICFEr em comparação com o valor de 50%, encontrado na população estadunidense.^6,20,21^

**Tabela 2:** – Comparação entre a coorte estudada e o registro ADHERE

	COORTE	ADHERE
**Mortalidade Intrahospitalar**	15,1%	4,1%
Idade, anos	63,04 (±14,3)	72 (±13,9)
Sexo masculino (%)	56,6%	48%
IC isquêmica (%)	30,9%	59%
Insuficiência Renal (%)	21,7%	29%
FA / Flutter atrial (%)	30,6%	31%
IC conhecida (%)	75,7%	77%
Creatinina, mg/dL	1,4 (1,0-2,0)	1,8 (±1,7)
Sódio, mg/dL	136 (134-140)	138 (±5)
FC, bpm	81 (70-98)	N/A
PAS, mmHg	118 (103-135)	143 (±32,6)
PAD, mmHg	71 (62-85)	77 (±20)
Ureia, mg/dL	51 (34,9-83,5)	69 (±46,2)
Fração de Ejeção (%)	33 (24-45)	N/A
ICFER (%)	68,6%	56%

IC: insuficiência cardíaca; FA: fibrilação atrial; FC: frequência cardíaca; PAS: pressão arterial sistólica; PAD: pressão arterial diastólica; ICFER: IC de fração de ejeção reduzida.

O ID é a capacidade de um escore de detectar o grupo de pacientes que apresentará um determinado desfecho, evidenciado pela ASC da curva ROC. O escore testado demonstrou um ID modesto a bom para prever a MH em nossa população. Em nossa coorte, o ADHERE apresentou ID de 0,69, em comparação com 0,76 e 0,75 nos estudos originais, respectivamente. Esses valores estão de acordo com a maioria dos estudos com o mesmo propósito, que encontraram valores entre 0,60 e 0,89, sendo a maioria realizada em países desenvolvidos.^12^ Em uma coorte israelense com mais de três mil pacientes, a precisão do escore ADHERE foi baixa, com ASC de 0,59. No mesmo estudo, o escore *Get With the Guidelines – GWTG,* também proposto pela Sociedade Americana de Cardiologia, teve um bom desempenho, com ASC de 0,75.^14^

É interessante notar que, embora vários estudos tenham avaliado a validação externa de escores prognósticos, o impacto dessa estimativa de risco nos desfechos dos pacientes hospitalizados por ICD não foi rotineiramente realizado.^12,20^ A maioria deles não avaliou a classificação, definida como a comparação entre o risco esperado e o encontrado, o que é necessário para a validação completa de um escore.^22^ Este é um passo importante quando se compara o resultado de uma instituição com o original. Em relação ao nosso resultado, embora a ureia pôde identificar pacientes de alto risco, como descrito no primeiro nó do escore ADHERE, nossa mortalidade foi significativamente maior do que a proposta pelo escore. Portanto, a classificação foi imprecisa. A subestimação do risco de mortalidade pelo escore, como observada em nossa coorte, poderia alocar pacientes em áreas de menor risco ou simplificar a monitorização e o tratamento, resultando em subtratamento e pior impacto nas taxas de sobrevida.

Por outro lado, embora as características demográficas e clínicas não sejam as mesmas entre as coortes, os fatores de risco para MH em pacientes cardiológicos podem ser semelhantes, como visto em um estudo que testou a precisão dos escores *ADHERE* e *GWTG-HF* em uma unidade de terapia intensiva, independentemente da patologia de admissão.^23^ Portanto, é crucial testar escores de risco bem conhecidos e validar seu uso em subpopulações específicas antes de desenvolver um novo escore. Variações dos escores de risco também podem ser feitas, adaptando-se às realidades ou objetivos locais. Uma coorte americana de ICD avaliou fatores que, além do escore ADHERE, poderiam identificar pacientes que necessitavam de vasopressores ou cuidados intensivos, uma condição descrita como IC piorada. Após a validação, eles encontraram um ID de 0,72.^24^ Estudo brasileiro que estudou coorte entre 2013 e 2020 encontrou dados semelhantes, com um ID de 0,66.^25^

Nosso estudo tem algumas limitações. Primeiro, o estudo foi conduzido durante a pandemia de Covid-19, o que pode ser uma limitação importante devido ao viés de confusão, uma vez que a mortalidade provavelmente foi mais alta nesse período. Pacientes com Covid-19 não foram excluídos da análise. Foi um estudo retrospectivo e, por isso, houve dados ausentes. Mais de 10% dos nossos pacientes não puderam completar o escore. A avaliação unicêntrica pode ser considerada um viés, sobretudo quando a generalização dos resultados é pretendida. Para utilização dos resultados encontrados em população brasileira, seria necessária avaliação de uma amostra mais representativa da população.

## Conclusão

O escore de risco ADHERE não pôde ser validado para prever a mortalidade hospitalar em nossa população de pacientes com insuficiência cardíaca aguda. A ureia sérica foi o único fator isolado que se correlacionou com o desfecho. O uso de escores de risco não testados em populações locais pode levar à subestimação ou superestimação do risco, o que tem implicações no manejo e alocação de recursos. A validação externa e a adaptação de escores à população local são essenciais.
