# Supplementary material for: Avaliação da Acurácia do Escore ADHERE em uma Coorte Brasileira Internada por Insuficiência Cardíaca
Source: Arq Bras Cardiol. 2025 Dec 18;122(11):e20250031. [Article in Portuguese] doi: 10.36660/abc.20250031 (PMC12978283; doi:10.36660/abc.20250031)
Supplement: Translation

# Translation: Assessment of the Accuracy of the ADHERE Score in a Brazilian Acute Heart Failure Cohort

**DOI:** 10.36660/abc.20250031i

**Authors:** Bruno Reznik Wajsbrot, Ana Luiza Ferreira Sales, Andre Luis Sales Feitosa, Carolina Pereira de Barros, Daniel Xavier de Brito Setta, Felipe Neves de Albuquerque, Marcelo Imbroinise Bittencourt, Pedro Pimenta de Mello Spineti, Simone Offrede Rego, Denilson Campos de Albuquerque, Roberto Esporcatte, Ricardo Mourilhe-Rocha

**Affiliations:** 1 Brazil Universidade do Estado do Rio de Janeiro, Rio de Janeiro, RJ – Brazil; 2 Brazil Hospital Pró-Cardíaco, Rio de Janeiro, RJ – Brazil; 3 Brazil Hospital Unimed-Rio, Rio de Janeiro, RJ – Brazil; 4 Brazil Complexo Hospitalar Américas – Hospitais Vitória e Samaritano Barra, Rio de Janeiro, RJ – Brazil

**Keywords:** Heart Failure, Prognosis, Mortality

## Abstract

**Background:**

Patients hospitalized for acute heart failure present a high mortality rate, so risk stratification for in-hospital death is of utmost importance. Risk scores can help physicians to identify patients at higher risk, but it is important to validate those scores in different populations, since sociodemographic characteristics may be very heterogeneous.

**Objectives:**

This study aimed to evaluate the ADHERE (Acute Decompensated Heart Failure Registry) risk score in a Brazilian quaternary teaching care center.

**Methods:**

This was a retrospective cohort study involving 304 patients with acute heart failure aged ≥ 18 years old, conducted between September 2019 and July 2022. The primary endpoint was the evaluation of the ADHERE score by analysis of the discriminatory index and classification. The secondary endpoint was the evaluation of other prognostic factors for in-hospital mortality. A p-value < 0.05 was considered statistically significant.

**Results:**

ADHERE score presented a discriminatory index of 0.69. ADHERE classification index was suboptimal, since the score did not stratify mortality risk on five strata as proposed originally. Furthermore, the score underestimates risk in the studied population. Admission serum urea was the only isolated prognostic factor for the endpoint (OR 1.043; CI 95% 1.024-1.062; p<0.001).

**Conclusion:**

The ADHERE risk score cannot be entirely validated in our cohort, since classification was not achieved despite an ideal discriminatory index. Admission serum Urea was the only independent risk factor associated with in-hospital mortality. Our study emphasizes the importance of correct external validation of a prognostic score, especially if the demographic and clinical characteristics of cohorts are not comparable.

## Introduction

Cardiovascular disease is the major cause of premature death in the world.^1^ Heart failure (HF), one of the main representatives of the group, can be considered a noninfectious epidemic disease,^2^ as it affects about 26 million people worldwide.^3^ Over the last ten years in Brazil, cardiovascular diseases were the second most prevalent cause of death, and HF is one of the main specific causes present in this group.^4^

Acute heart failure (AHF) is a severe complication of the syndrome and is defined by the European Society of Cardiology as “rapid or gradual onset of symptoms and/or signs of HF, severe enough for the patient to seek urgent medical attention”.^5^ In-hospital mortality (IHM) can be as high as 20%, depending on the patient’s characteristics and the severity of the sharpening.^6^ The median IHM in Europe and the U.S. is 4.9%^7^ and 4.0%,^6^ respectively, while the biggest cohort of AHF in Brazil (BREATHE study) showed an IHM of 12.6%.^8^ In order to predict IHM for patients hospitalized for AHF, the 2022 American Heart Association Guideline for the Management of Heart Failure^9^ suggests the use of the ADHERE (Acute Decompensated Heart Failure Registry) score.^6^

Nonetheless, there is great variability in sociodemographic characteristics between different populations, and prognostic factors might vary between groups. For example, isolated hypertension not associated with ischemic heart disease (IHD) is highly associated with HF in Sub-Saharan Africa and Latin America, but not in developed countries, where ischemic etiology prevails.^10^ On the BREATHE study (I Brazilian Registry of Heart Failure), the prevalence of ischemic cardiomyopathy in Brazil ranges from 16.5% to 33.6%, depending on the region studied^8^. Mortality risk scores should be able to discriminate the severity of an illness, allowing the correct allocation of resources during treatment. Nevertheless, for the best achievement of such an objective, external validation is imperative^11^ (Central Illustration).

Although cardiology societies encourage the use of the ADHERE score on AHF patients, complete validation using all statistical criteria is rarely done.^12-14^ This study aimed to evaluate the performance of the ADHERE risk score on a Brazilian population from a public quaternary teaching care center. Secondary objectives were the evaluation of demographic and clinical data and their relationship to IHM.

## Methods

This was a single-center retrospective longitudinal study. Data was collected from electronic medical records between September 2019 and July 2022. Patients admitted with a diagnosis of AHF according to the 2021 definition of the European Society of Cardiology^5^ were included. Only the first patient admission was analyzed. Exclusion criteria were age under 18 years, previous diagnoses of malignancy in palliative care, or patients on renal replacement therapy.

All participants underwent demographic and clinical assessment at admission, in addition to heart rate and arterial blood pressure. Laboratory data, including serum sodium, serum potassium, serum hemoglobin, serum urea, serum creatinine, serum random glucose, and serum glycated hemoglobin, were included. Renal failure was considered when estimated glomerular filtrated rate (e-GRF) was under 60ml/min/m^2^.^15,16^ The presence of diabetes mellitus was considered if self-reported or if the patient had a random glucose ≥200mg/dL or glycated hemoglobin ≥6,5%.^17^ Echocardiographic parameters were extracted from the first exam performed after admission or a previous one, if there was no information from an in-hospital echocardiogram. Parameters evaluated were ejection fraction, qualitative description of left and right ventricular function, end diastolic and end systolic left ventricular diameter, left atrium volume, and presence and quantification of aortic and mitral regurgitation.

### Statistical analysis

The Kolmogorov-Smirnov test was performed to assess the normality of the distribution of continuous variables. For descriptive analyses, continuous variables were expressed as means (standard deviation) for normally distributed data, medians (interquartile range) for asymmetrically distributed data, or absolute numbers (proportions) for categorical data. Differences between the two groups were examined by the unpaired T-test or Mann–Whitney test for normal variables and non-parametric variables, respectively, and by Chi-Squared test or Fisher’s exact tests for categorical variables. A multivariate logistic regression analysis was performed to investigate variables independently associated with in-hospital death. A Kaplan-Meier curve with a log-rank test was used for survival analysis. Score validation was done by analysis of the discriminatory index (DI) and calibration.^18^ Logistic ADHERE score using the equation published on the original data was used for analysis of the AUC of the ROC curve, which is the DI. Calibration was done by comparing expected and observed events in the Brazilian cohort. All tests were bicaudal, and p<0,05 was considered statistically significant. Analyses were performed with SPSS software 21.0 (Chicago, USA).

The study was reviewed and approved by the Health Research Ethics Committee, Faculty of Medical Sciences, State University of Rio de Janeiro, number 3.706.949.

## Results

A total of 458 admissions described as myocardiopathy (ICD 25.5), non-specified HF (ICD 50.9), and congestive HF (ICD 50.0) were enrolled. Of those, 154 were excluded because they were repeated admissions of the same patient, resulting in 304 admissions analyzed.

Demographic, clinical variables, echocardiographic, and laboratory data can be found in Table 1. The mean age was 63.04 years (SD±14.3). 56.6% were male, 65.8% had arterial hypertension, 33.3% diabetes mellitus, 30.6% had a previous diagnosis of atrial fibrillation or flutter, and 30.9% had IHD; 75.7% were classified as worsening HF and 24.5% as acute onset HF. The ejection fraction was measured in 274 patients (90.1%). Of them, 188 (68.6%) were classified as HF with reduced ejection fraction (HFrEF), 37 (13.5%) as HF with mildly reduced ejection fraction (HFmrEF), and 49 (17.9%) as HF with preserved ejection fraction (HFpEF). 156 (52.0%) had associated right ventricular dysfunction. IHM was 15.1% (46/304) for the entire cohort and 18.4%, 16.2% and 13.3% for the subgroups of HFpEF, HFmrEF, and HFrEF, respectively (p=0.64).

**Table 1:** – Demographic, clinical variables, echocardiographic, and laboratory data of the cohort and subgroups

Variable	Total	Discharge	Death	p-value
	n=304	n=258 (84.9%)	n=46 (15.1%)	
Age, Years	63.04 (±14.3)	62.22 (±14.1)	67.59 (±15.07)	0.02
Male, n (%)	172 (56.6%)	152 (58.9%)	20 (43.5%)	0.052
Ischemic HF, n (%)	92 (30.9%)	80 (31.5%)	12 (27.3%)	0.724
Hypertension, n (%)	198 (65.8%)	167 (65.2%)	31 (68.9%)	0.734
DM, n (%)	102 (33.7%)	81 (31.9%)	20 (43.5%)	0.126
Renal Failure, n (%)	65 (21.7%)	54 (21.2%)	11 (24.4%)	0.624
AFib or Flutter, n (%)	92 (30.6%)	74 (28.9%)	18 (40%)	0.136
COPD, n (%)	19 (6.3%)	18 (7%)	1 (2.2%)	0.327
Smoking, n (%)	58 (19.1%)	52 (20.2%)	6 (13%)	0.313
Alcoholism, n (%)	38 (14.6%)	33 (14.9%)	5 (12.5%)	0.811
Previous HF, n (%)	181 (75.7%)	153 (74.6%)	28 (82.4%)	0.394
Hemoglobin, mg/dL	13 (11.4-14.5)	13 (11.5-14.4)	12.1 (10.5-14.8)	0.429
Creatinine, mg/dL	1.4 (1.0-2.0)	1.38 (1.03-1.09)	1.7 (1.2-2.7)	0.003
eGFR (ml/min/m^2^)	47 (23-75)	48 (24-76)	34 (16-71)	0.016
Sodium, mg/dL	136 (134-140)	137 (134-140)	135 (131-138)	0.008
Potassium, mg/dL	4.3 (3.8-4.8)	4.3 (3.8-4.8)	4.2 (3.8-4.8)	0.68
Heart Rate, bpm	81 (70-98)	81 (70-98)	82 (74-94)	0.807
SBP, mmHg	118.5 (103-135)	120 (107-140)	110 (100-130)	0.037
DBP, mmHg	71 (62-85)	74 (63-85)	70 (60-80)	0.017
Urea, mg/dL	51 (34.9-83.5)	49.0 (34-76.9)	83 (39.8-145.9)	0.001
LV ejection fraction, %	33 (24-45)	33 (24-45)	35 (25-48.7)	0.585
LV EDD, mm	59 (±11.8)	59.3 (±11.14)	57.6 (±15.27)	0.503
LV ESD, mm	49 (39-58)	49 (40-58)	48.5 (32.2-58)	0.574
LA volume, mL/m^2^	53 (32.2-64)	51 (42-62.5)	59 (53.5-73.7)	0.001
Severe MR, n (%)	49 (19.2%)	41 (18.9%)	8 (21.1%)	0.755
RV dysfunction, (%)	156 (52.0%)	128 (50.4%)	28 (60.9%)	0.203

IQR: interquartile range; HF: heart failure; DM: diabetes mellitus; AFib: atrial fibrillation; COPD: chronic obstructive pulmonary disease; e-GFR: estimated glomerular filtration rate; SBP: systolic blood pressure; DBP: diastolic blood pressure; LV: left ventricle; LV EDD: left ventricle end diastolic diameter; LV ESD: Left ventricle end systolic diameter; LA: left atrium; MR: mitral regurgitation; RV: right ventricle.

### Primary endpoint

ADHERE could be calculated in 87.5% (266/304) of the patients. The DI of the score showed an AUC of 0.69 (Figure 1). ADHERE classification was inadequate to predict IHM in the five risk groups proposed. Only the first split, represented by admission serum urea, could create two nodes of different IHM (OR 4.45; CI 95% 2.29-8.65; p<0.001). The second ADHERE predictor, represented by systolic blood pressure, did not classify subgroups that were proposed originally. Classification analysis can be found in Figure 2.

**Figure 1:** – Discriminatory index of ADHERE score. Source: the author.

**Figure 2:** – Classification analysis of ADHERE score. SBP: systolic blood pressure. Source: the author.

### Secondary endpoints

Variables associated with IHM on univariate analysis were older age (p=0.02), lower systolic blood pressure (p=0.037), lower diastolic blood pressure (p=0.017), higher BUN (p=0.001), higher creatinine (p=0.003), lower serum sodium (p=0.005), and higher left atrium volume (p=0.008). An exploratory model with binary logistic regression with age, SBP, BUN, e-GFR, and serum sodium showed that only BUN was independently related to IHM (OR 1.043; CI 95% 1.024-1.062; p<0.001). The DI of admission urea was 0.65. The first split of the ADHERE score, represented by urea values above or below 92mg/dL, could stratify the population into subgroups that had IHM of 33.3% and 10.1%, respectively.

## Discussion

Our study aimed to evaluate the performance of the ADHERE risk score in a specific Brazilian quaternary teaching hospital. This study has one main finding. The proposed score was inaccurate and could not be entirely validated to predict IHM in this specific population. So, the main message is that validation of risk scores before application to clinical practice is imperative. Although suggested by guidelines, they often are inaccurate when used on different populations.

Our cohort is drawn from a quaternary teaching hospital within the Brazilian public health care system, specialized in the treatment of patients with advanced HF. In contrast, the ADHERE registry includes data from over 65,000 patients admitted to 263 U.S. hospitals between 2001 and 2003. Our cohort likely represents a population with more severe disease compared to both American and other Brazilian centers. Notably, the IHM observed in the BREATHE^8^ study was lower than in our cohort, despite including non-specialized centers, suggesting that our sample comprised patients with more advanced disease and distinct clinical characteristics. Therefore, generalizing our findings to the broader Brazilian population is not feasible.

Comparison between our cohort and the ADHERE registry can be found in Table 2. Our cohort is almost ten years younger, had lower admission blood pressure values, and lower serum urea and creatinine than the population from the ADHERE study. Diabetes and hypertension were also less well-known. Distribution of HF phenotypes might also be different between cohorts.^19^ We had more than 70% of HFrEF in comparison to half of the population from American´s.^6,20,21^

**Table 2:** – Comparison between our Brazilian cohort and the ADHERE registry

	COHORT	ADHERE
**In-hospital mortality**	15.1%	4.1%
Age, years	63.04 (±14.3)	72 (±13.9)
Male gender (%)	56.6%	48%
Ischemic HF (%)	30.9%	59%
Renal failure (%)	21.7%	29%
Flutter / AFib. (%)	30.6%	31%
Known HF (%)	75.7%	77%
Creatinine, mg/dL	1.4 (1.0-2.0)	1.8 (±1.7)
Sodium, mg/dL	136 (134-140)	138 (±5)
HR, bpm	81 (70-98)	N/A
SBP, mmHg	118 (103-135)	143 (±32.6)
DBP, mmHg	71 (62-85)	77 (±20)
Urea, mg/dL	51 (34.9-83.5)	69 (±46.2)
Ejection fraction (%)	33 (24-45)	N/A
HFREF (%)	68.6%	56%

HF: heart failure; AFib: atrial fibrillation; HR: heart rate; SBP: systolic blood pressure; DBP: diastolic blood pressure; HFrEF: heart failure with reduced ejection fraction.

DI is the ability of a score to detect the group of patients that will present a certain outcome, evidenced by the AUC of the receiver operating characteristic (ROC) curve. The tested score demonstrated a modest to good DI to predict IHM in our population. In our cohort, ADHERE had a DI of 0.69 compared to 0.76 and 0.75 on the original studies, respectively. Those values are in accordance with most studies that had the same purpose, which found values between 0.60 and 0.89, most of them performed on developed countries.^12^ In a single-center study with more than six thousand admissions in the USA, ADHERE had a DI of 0.66 and GWTG a DI of 0.74 to predict IHM. In an Israeli cohort with more than three thousand patients, the accuracy of the ADHERE score was low, with an AUC of 0.59. In the same study, GWTG performed well, with an AUC of 0.75.^14^

It is interesting to notice that although several studies evaluated external validation of prognostic scores, the impact of this risk estimation on the outcomes of patients hospitalized for AHF was not routinely performed.^12,20^ Most of them did not evaluate classification, defined as comparison between expected and found risk, which is necessary for the complete validation of a score.^22^ This is an important step when a specific institution’s result is compared to the original one. Although urea could detect high-risk patients in our cohort, as described on the first node of ADHERE, our mortality was significantly higher than proposed by the score, invalidating the use in clinical practice, as it will underestimate mortality risk. Therefore, the classification was inaccurate. Underestimation of a mortality risk, as observed in our cohort, could allocate patients to open yards or simplify monitoring and therapy, generating undertreatment with a worse impact on survival rates.

On the other hand, although demographic and clinical characteristics are not the same between cohorts, risk factors for IHM in cardiologic patients might be similar, as we can see in a study that tested the accuracy of ADHERE and GWTG-HF scores on an intensive care unit, independently of admission pathology.^23^ So, it is crucial to test well-known risk scores and validate their use in specific subpopulations before developing a new one. Variations of risk scores could also be done, adapting to local realities or objectives. An American cohort of AHF evaluated factors that, in addition to the ADHERE score, could identify patients who need vasopressors or intensive care unit, a condition they described as worsening HF. After validation, they found a DI of 0.72.^24^ A Brazilian study that studied a cohort between 2013 and 2020 found similar data, with an ID of 0.66.^25^

Our study has some limitations. First, the study was conducted during the COVID-19 pandemic, and this could be an important limitation because of confusion bias, as mortality was probably higher during that period. COVID-19 patients were not excluded from the analysis.

It was a retrospective study, and because of that, we had some missing data. More than ten percent of our patients could not complete the score. The single-center design of the study may be considered a limitation, particularly in the context of attempts to generalize the findings. To support broader applicability, a more representative sample of the Brazilian population should be investigated in future studies.

## Conclusion

The ADHERE risk score could not be validated to predict in-hospital mortality in our population of patients with acute heart failure. Serum urea was the only single factor that correlated with the outcome. The use of untested risk scores in local populations can lead to underestimation or overestimation of risk, which has implications for resource management and allocation. External validation and adaptation of scores to the local population are essential.
